# Lung nodule detection in chest X-rays using synthetic ground-truth data comparing CNN-based diagnosis to human performance

**DOI:** 10.1038/s41598-021-94750-z

**Published:** 2021-08-04

**Authors:** Manuel Schultheiss, Philipp Schmette, Jannis Bodden, Juliane Aichele, Christina Müller-Leisse, Felix G. Gassert, Florian T. Gassert, Joshua F. Gawlitza, Felix C. Hofmann, Daniel Sasse, Claudio E. von Schacky, Sebastian Ziegelmayer, Fabio De Marco, Bernhard Renger, Marcus R. Makowski, Franz Pfeiffer, Daniela Pfeiffer

**Affiliations:** 1 Chair of Biomedical Physics, Department of Physics and Munich School of BioEngineering, Technical University of Munich, 85748 Garching, Germany; 2 Department of Diagnostic and Interventional Radiology, School of Medicine and Klinikum rechts der Isar, Technical University of Munich, 81675 Munich, Germany

**Keywords:** Lung cancer, Computer science

## Abstract

We present a method to generate synthetic thorax radiographs with realistic nodules from CT scans, and a perfect ground truth knowledge. We evaluated the detection performance of nine radiologists and two convolutional neural networks in a reader study. Nodules were artificially inserted into the lung of a CT volume and synthetic radiographs were obtained by forward-projecting the volume. Hence, our framework allowed for a detailed evaluation of CAD systems’ and radiologists’ performance due to the availability of accurate ground-truth labels for nodules from synthetic data. Radiographs for network training (U-Net and RetinaNet) were generated from 855 CT scans of a public dataset. For the reader study, 201 radiographs were generated from 21 nodule-free CT scans with altering nodule positions, sizes and nodule counts of inserted nodules. Average true positive detections by nine radiologists were 248.8 nodules, 51.7 false positive predicted nodules and 121.2 false negative predicted nodules. The best performing CAD system achieved 268 true positives, 66 false positives and 102 false negatives. Corresponding weighted alternative free response operating characteristic figure-of-merits (wAFROC FOM) for the radiologists range from 0.54 to 0.87 compared to a value of 0.81 (CI 0.75–0.87) for the best performing CNN. The CNN did not perform significantly better against the combined average of the 9 readers (*p* = 0.49). Paramediastinal nodules accounted for most false positive and false negative detections by readers, which can be explained by the presence of more tissue in this area.

## Introduction

With accounting for over 1.7 million deaths in 2018, lung cancer is one of the most common causes of cancer death worldwide^1^. Regular screening using chest x-ray (CXR) or low dose computed tomography (LDCT) is under investigation, with the latter being more effective, but also more expensive^2–4^. While standard CXR screening has only shown to improve early detection but not a decrease in mortality^2^, computer aided diagnosis (CAD) systems could increase sensitivity and therefore improve its benefit as a screening method^3^.

While the applied dose for CXR is significantly lower than for CT (typically 0.1 mSv for a posteroanterior and lateral CXR study and 1.5 mSv for low dose CT)^5, 6^, the detection of nodules in chest CXR is more challenging than for CT. Lung metastases often originate from extra-thoracic malignancies (ETM), with the lungs being a frequent site of metastatic growth: for patients, who died of an ETM, incidences of pulmonary metastases are reported to be greater than 19%^7–9^.

Hence, it is of interest to identify positions in the lung, where radiologists have problems detecting nodules correctly in order to improve training.

Additionally, radiologists can be assisted by CAD systems for CXR diagnosis^10, 11^ and it may further improve sensitivity for CXR based lung cancer screening^3^. Here, with the rise of computing power, deep-learning based CAD systems gained interest recently: for automatic x-ray image classification several approaches have been published^12–15^, which may assist radiologists in clinical practice. U-Net like architectures were successfully employed for segmentation tasks^16, 17^ and RetinaNet based detector for object detection tasks in radiographs^18–21^. While U-Net based implementations yield a segmentation as output, RetinaNet detectors output bounding boxes which most likely contain the object of interest (in our case a nodule). Additional scores for each box reflect the certainty of the network for a detection here. Both approaches require either pixel-level or bounding-box annotations for training and evaluation of deep-learning systems.

Such annotations are cost expensive, as annotations usually have to be carried out by an expert in the respective area. In our case, for CXR nodule detection, a radiologist needs to mark suspected lesions by hand in order to make the data applicable for CNN training and evaluation. Therefore, an option to run pre-clinical trials is the use of synthetic data. Here, virtual clinical trials (VCTs) play an important role in the testing of imaging systems^22^. In these trials, usually the body anatomy and physics of the image acquisition system are simulated. Such systems have been developed for a wide range of modalities, such as CT^23^ or mammography^24^. However, the use of computational phantoms does never completely resemble a real human anatomy.

For a lung nodule detection task, it is possible to combine a real anatomy by the use of real CT scans with synthetic (or virtual) nodules: Yu et al. developed a simulation framework for nodule detection in CT scans where a virtual nodule was inserted into a real scan and nodule detection performance was evaluated by 4 observers^25^. For lung nodule detection in radiographs our simulation approach is very similar, but additionally generates a radiograph from the CT scan: we place nodules in random positions within the lung of a CT scan and forward project the CT scan. Hence, it is possible to generate a lot of different radiographs for each available CT scan by altering the nodule positions. Contrary to manually annotated data, it is possible to retrieve the exact contours of every inserted tumor (e.g. the groundtruth), which is beneficial for training CNNs with box annotations or pixel-level annotations.

In this study, the usability of simulated CXRs for lung nodule detection performance evaluation and CNN training is demonstrated. We train multiple CNNs with synthetically generated data and evaluate the performance against nine radiologists. It is shown that on synthetic data, CNNs are able to reach a performance similar to radiologists. The simulation framework further allows to examine the areas of false negative detections, e.g. areas where radiologists had problems identifying tumors.

## Results

**Figure 1:**
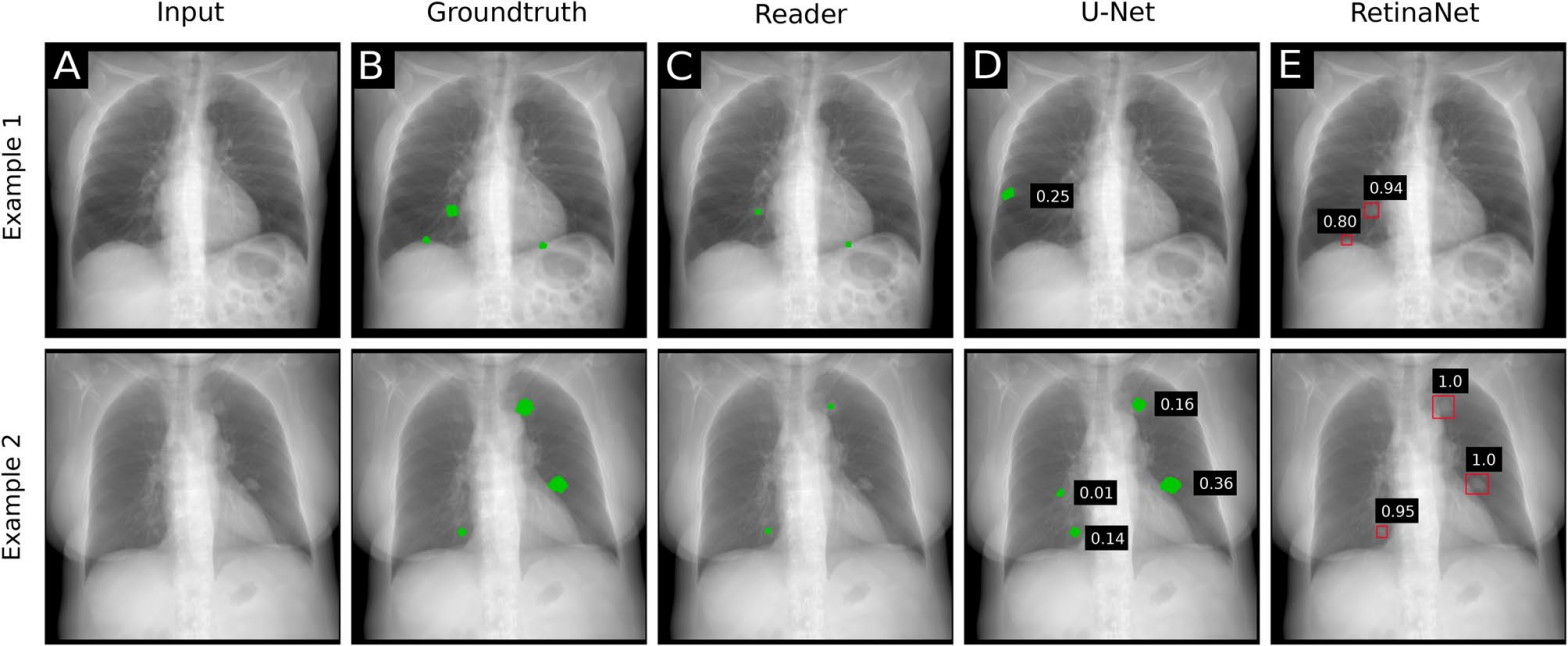
Synthetic radiographs, ground truth masks and results of reader and computer-based detection. (**A**) Synthetic input radiograph as shown to the reader and evaluated by the CNNs (**B**) corresponding ground-truth radiograph with nodules marked green (**C**) center position of nodules marked by a reader (**D**) U-Net prediction (**E**) RetinaNet bounding-box predictions with scores.

**Figure 2:**
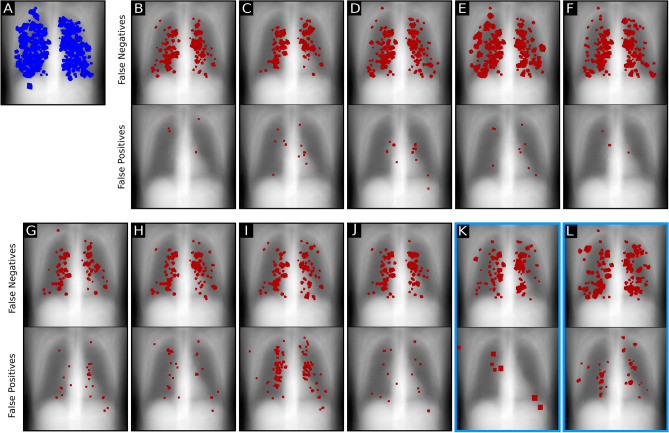
Localization of false negative and false positive predictions in the reader study. Backgrounds were determined by averaging over all reader study radiographs. (**A**) All inserted nodules of different sizes in all radiographs marked blue. (**B**–**J**) False negative and false positive predictions by reader. (**K**) Location of false negative predictions of RetinaNet and false positive predictions of RetinaNet. (**L**) False negative predictions of U-Net and false positive predictions of U-Net.

The performance of nine radiologists and two CNN algorithms was evaluated for the nodule detection task. Example detections are shown in Fig. 1. False negative detections are shown in Fig. 2. Absolute true positive, false positive and false negative numbers are reported in Table 1.

**Table 1:** True positives (TP), false positives (FP) and true negatives (FN) for RetinaNet, U-Net and the readers. For RetinaNet a nodule with a confidence score greater than 0.5 was counted as positive.

	TP	FP	FN
RetinaNet	268	66	102
U-Net	256	279	114
Reader 1	244	5	126
Reader 2	278	15	92
Reader 3	207	29	163
Reader 4	185	9	185
Reader 5	201	9	169
Reader 6	294	35	76
Reader 7	273	52	97
Reader 8	281	276	89
Reader 9	276	35	94

Here, U-Net yielded a high rate of true positives, but also the most false positives. To retrieve a combined score of false positives and true positives, weighted alternative free response operating characteristics (wAFROC) FOMs were calculated and presented in Table 2.

**Table 2:** Figure of merits (FOM), 95% confidence intervals (CI) and standard error (StdErr) for wAFROC metrics. The weighted lesion localization fraction (wLLF score) was retrieved at an x-axis 0.2 operating point for all readers and CNNs on the wAFROC curve.

	wAFROC				
	FOM	CI Lower	CI Upper	StdErr	wLLF
RetinaNet	0.81	0.75	0.87	0.028	0.71
U-Net	0.58	0.47	0.68	0.052	0.41
Reader 1	0.82	0.79	0.86	0.017	0.71
Reader 2	0.87	0.83	0.90	0.017	0.79
Reader 3	0.74	0.68	0.79	0.029	0.57
Reader 4	0.74	0.70	0.78	0.022	0.59
Reader 5	0.78	0.75	0.81	0.015	0.65
Reader 6	0.87	0.83	0.91	0.020	0.79
Reader 7	0.83	0.79	0.88	0.022	0.74
Reader 8	0.54	0.44	0.63	0.048	0.31
Reader 9	0.84	0.79	0.88	0.023	0.75

**Figure 3:**
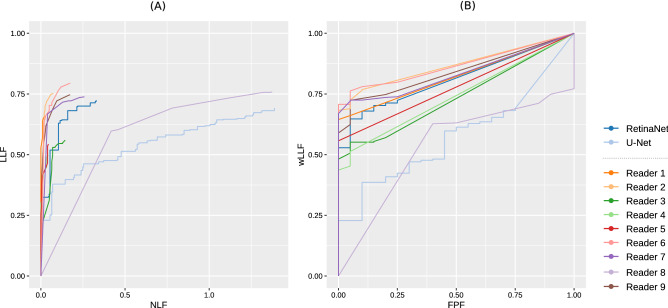
Comparison of CNN and reader based diagnostic performance. (**A**) FROC plot with lesion localization fraction (LLF) plotted against non lesion fraction (NLF) (**B**) wAFROC plot with weighted LLF on the ordinate. The plot was generated using RJafroc^26^.

**Figure 4:**
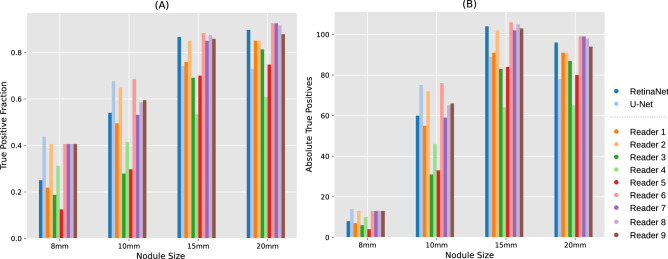
Comparision of detection performance by nodule size (**A**) Relative true positive fraction (**B**) absolute number of true positives for RetinaNet CNN, U-Net CNN and readers R1–R9. The plot was generated using Matplotlib^27^.

The FOM score of the RetinaNet network was higher than that of four readers and lower than that of five readers. The FOM score of U-Net was lower than that of eight readers. Corresponding graphs are shown for FROC (Fig. 3A) and wAFROC (Fig. 3B) metrics. The RetinaNet CNN did not perform better against the average of all readers (0.78 average reader FOM, *p* = 0.49). Combining U-Net and RetinaNet, by counting the bounding boxes of RetinaNet as positive when a U-Net segmentation was found within the bounding-box area, the FOM score decreases slightly to 0.78. True positives with respect to nodule size are shown in Fig. 4.

## Methods

**Figure 5:**
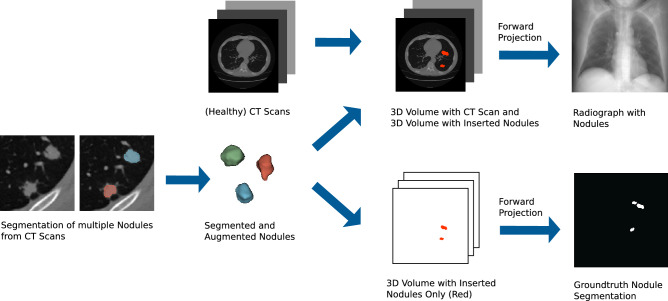
Workflow for generating synthetic radiographs containing tumour nodules with perfect ground truth knowledge. Based on natural shapes, various sizes of tumors are generated and subsequently inserted into clean CT scans and different locations. The 3D CT data set is then forward projected to generate p.a. thorax radiographs. In parallel, the tumors only are forward projected to obtain perfect ground-truth masks. These ground-truth masks is later used to compare the radiologist’s findings with the expected findings.

Radiographs for network training and validation (U-Net and RetinaNet) were generated from 855 CT scans of a public dataset. For the reader study, radiographs were generated from nodule-free CT scans with altering nodule positions, sizes and nodule counts per radiograph. Nodules were segmented from another CT scan, augmented and inserted into each of the CT scans at a randomly selected position within the lung. Next, a forward projection was performed in order to generate a realistic, synthetic radiograph. By changing nodule position, size and count, this technique allows the generation of multiple, different radiographs out of a single CT scan. Overall workflow is illustrated in Fig. 5.

### Preprocessing

To simulate a x-ray image, HU values are converted back to their respective absorbtion values. The absorbtion value for a voxel $\mu _x$, can be calculated from a HU voxel value $HU_x$ according to:

$$\begin{aligned} \mu _x = \mu _{water} + (\mu _{water} - \mu _{air}) \cdot \frac{HU_x}{S}, \end{aligned}$$

whereat the scale factor *S* is vendor specific and usually 1000 or 1024 and $\mu _{water}$=$2.059 \times 10^{-1}\,\text {cm}^{-1}$ . A first step is to remove the patient table from the CT scan, as the table does not appear in radiographs. This is done by using a combination of thresholding and a connected component algorithm, which removes the second largest object (table). To segment the lung, a tissue mask around the lung is extracted by thresholding. Afterwards the lung-area is identified using a hole-filling algorithm.

### Nodule insertion

During training, in each CT scan between 1 and 6 nodules are inserted. Each nodules is chosen from 19 segmented nodules with an equal probability. The nodule is normalized to values between 0 and 1 and multiplied with the absorption value of soft tissue. Furthermore the nodule is randomly augmented during the training process: Here, the nodule is rotated on the coronal plane by a value chosen from a uniform distribution between 0 and 360 degrees. Furthermore, the nodule is randomly scaled to values between 8 and 20 mm along all three axes. The nodule-free volume and the volume with the nodules are forward projected and summed up in order to obtain a simulated diseased radiograph, whereby absorption coefficients are weighted by their voxel size. Also the nodules without the surrounding CT volume are forward projected in order to obtain the groundtruth. Both the diseased radiograph and the groundtruth are resized to 512 $\times$ 512 pixels for training.

### Dataset description

Data access was approved by the institutional ethics committee at Klinikum Rechts der Isar (Ethikvotum 87/18 S) and the data was anonymized. The ethics committee has waived the need for informed consent. All research was performed in accordance with relevant guidelines and regulations.

**Figure 6:**
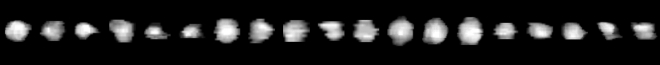
Projected segmentations of 19 nodules, which were artificially inserted into the synthetic radiographs.

CNN training was performed with 855 CT scans from the LUNA16^28^ dataset, whereat 80% of data was chosen for training and 20% of data for validation. Nodules to be inserted in the CT scans were segmented from 5 CT scans. Segmented lung nodules were metastasis from various malignant tumors. Metastases origin tumor was carcinoma of the breast in two patients (9 metastases total), colorectal cancer in two patients (5 metastases total) and melanoma in one patient (5 metastases total). Projected segmentations are shown in Fig.  6.

For the reader study, a dataset of 21 CT scans was collected from our institution’s picture archiving and communication system (PACS). These scans were checked to be unsuspicious (nodule-free) by one radiologist (JB, 3 years of experience).

### Reader study

For the reader study, 201 radiographs were generated from 21 nodule-free CT scans with altering nodule positions, sizes and nodule counts per radiograph. Diameters for inserted nodules in the reader study were 8 mm, 10 mm, 15 mm and 20 mm. Of all radiographs, 20 radiographs contained no nodule, 53 radiographs contained 1 nodules, 67 radiographs contained 2 nodules and 61 radiographs contained 3 nodules. Within the nodule-present cases, corresponding fractions are 53/181 cases with one nodule, 67/181 cases with 2 nodules and 61/181 cases with three nodules.

Of 370 inserted nodules, 32 had a size of 8 mm, 111 had a size of 10 mm, 120 had a size of 15 mm, and 107 had a size of 20 mm.

Reader experience was one month for one radiologist, nine month for one radiologist, at least one year for two radiologists, at least two years for two radiologists and at least three years for three radiologists. Readers were given the task of marking lung tumors and indicating confidence for each tumor on a scale from 1 to 100. Only posterior-anterior radiographs were used in the reader study. In order to simulate a clinical setting, each radiologist was given a time constraint of 20 seconds per radiograph.

The reader study dataset was also the test set for CNN evaluation in order to compare CNN performance to reader performance. It was ensured, no CT scans of the test set or the reader study were part of the training or validation set.

### CNN architectures and network training

**Figure 7:**
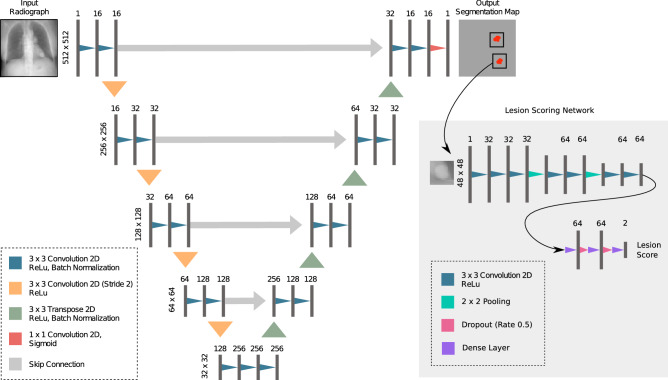
Architecture of the utilized U-Net. Input and output were a single channel 512 $\times$ 512 matrix. Downsampling was performed using convolutional layers with a stride of 2. A second network (lesion scoring network) was used to retrieve a per-lesion score of the segmented nodules. Numbers above layers indicate convolution filters for convolution layers and number of neurons for dense layers.

We investigate two CNN architectures: First, a U-Net^17^ like architecture is used and second, a RetinaNet^20^ based object detector is trained. For the U-net architecture, training was performed for 400 epochs with 3200 steps per epoch and a batch size of 1. Adam optimizer parameters were set to $\beta _1 = 0.9$ and $\beta _2 = 0.999$ with a learning rate of $10^{-3}$. Applied loss function was a Dice loss, as suggested by Milletari et al.^29^. In order to retrieve per lesion score for the U-Net, we trained a second helper network for the U-Net: A lesion scoring network, which inputs a patch centered on the lesion, was trained for 500 epochs with a learning rate of $10^{-5}$, a batch size of 32 and 87 steps per epoch. Augmentation included rotation, shift and flip operations. Positive and negative patches were equally sampled. Positive patches for the lesion scoring network were extracted from the available training segmentations. Using a hard-negative mining^30, 31^ approach, negative patches for the lesion scoring network were extracted from positions, where the U-Net yielded a prediction on healthy radiographs. The overall architecture for the U-Net based approach is illustrated in Fig 7.

For RetinaNet, training was performed for 50 epochs with a step size of 1000. The batch size was set to 1. The backbone was set to ResNet-101^32^. Loss function hyperparameters were set to $\alpha = 0.25, \gamma = 2.0$. The learning rate was set to $10^{-5}$. It was reduced by factor 0.1 after the loss did not change for more than 3 epochs ($\delta = 0.0001$). Data augmentation transformations for RetinaNet included contrast, brightness, shear, scale, flip, and translation. Models were implemented using Tensorflow^33^ and Keras^34^. Plots were generated using Matplotlib^27^ and RJafroc^26^. Furthermore, RetinaNet models are based on *keras-retinanet*^35^. Weights were obtained from the epoch with the best validation loss for both architectures.

### Data analysis

Usually the tradeoff between sensitivity and specificity can be analyzed using receiver operating characterisics (ROC). As this technique is only applicable for binary classification tasks on case-level, free response ROC (FROC) methods to evaluate detection performance on lesion level were introduced^36–38^. Here, a FROC plot consists of two axes: (1) The lesion localization fraction (LLF), defined as the number of true positives divided by the total number of lesions. (2) The non lesion localization fraction (NLF) as the total number of false positives divided by the total number of cases. However, in this method patients with more lesions are weighted more. To compensate for this, the weighted alternative FROC (wAFROC)^38–40^ is used: it assigns a weight *w* to each lesion, which sum up to unity on patient level and therefore ensures each patient is an equal representative of the population.

Given a threshold $\zeta$, above which nodules are counted as positives, the number of nodule-containing cases $K_N$, the number of Lesions $L_k$ for each case k, the lesion weight $W_{k l}$ and the indicator function *I*, which returns 1 if the argument is true and zero otherwise,

$$\begin{aligned} wLLF_r(\zeta ) = \frac{1}{K_N} \sum _{k=1}^{K_N} \sum _{l=1}^{L_k} W_{k l} I (z_{k l } \ge \zeta ), \end{aligned}$$

and a false positive fraction (FPF)

$$\begin{aligned} FPF(\zeta ) = \frac{1}{K_F} \sum _{k=1}^{K_F} I (FP_k \ge \zeta ) , \end{aligned}$$

given the number of nodule-free cases $K_F$, which sums up nodule-free cases with false positive detections $FP_k$^39, 40^.

To count a prediction as true positive, a distance criterion needs to be defined. In our study, a true positive is counted if the distance of the prediction center of mass (COM) is below 30 pixels to the ground-truth and the score is above or equal $\zeta$. Furthermore, we define the weight *w* of all lesions in a single patient as equal (e.g. if a patient has 4 lesions, the weight of every lesion is 0.25). Software used for evaluation was RJafroc^26^. Significance testing was done with alpha = 0.05.

## Discussion

Research on nodule location detection in radiographs was primary performed using large sets of manually annotated data. To reach human observer performance, McKineey et al. reported 11734 annotated radiographs in their training dataset^18, 41^.However, it requires a lot of time and cost intensive work to annotate and delineate nodules. While approaches are available that work without pixel-level or box-level annotations, e.g. weakly supervised learning^12, 13, 15, 42, 43^ ,the provided output locations are usually not very accurate, compared to segmentation or bounding box approaches. Hence, the presented method is a potential alternative method for pre-clinical evaluation of deep learning systems without the need of large sets of manually annotated data. The benefit here is that, contrary to manually annotated data, the ground-truth delineations of tumors are perfectly accurate, and single tumors can not be missed or wrongly delineated.

However, the application of the trained model on non-simulated radiographs remains challenging and is still under development, referred to as domain randomization: Previous investigations tried to generate real-world car detection or robotic systems by use of synthetic images^44–46^. This can be achieved by applying a large amount of unrealistic pertubations to the training domain. Studies in the medical field were performed by Toth et al.^47^, who registered cardiac models to radiographs. In our work we already did first preparations to transfer the CT generated data to the CXR domain by removing the patient table from the CT scan. Further challenges are the different arm positions in CT scans and higher resolution of radiographs. The arms can not be simply removed or masked out, as the different pose affects the position of the scapula and thus the visibility of the lungs is different for the two modalities.

To scale up resultion a superresolution network like Yamanaka et al.^48^ can be applied. Superresolution networks already have been applied successfully to chest-radiographs^49^. However, in our opinion, the main challenge lies in the modelling of the tumor shape. In this work, we used a shape from a pool of 19 tumors, which is augmented by rotation and rescaling to model more tumor shapes. However, this approach does most likely not capture the complete variance of tumor shapes, as tumor shapes vary broadly. Here, a spatial tumor model as described by Vogelstein et al.^50^ could be helpful.

The employed framework facilitates to analyze locations of false negative detections, and doing so showed some differences between CNN and radiologists: While most false negative detections by radiologists and by the RetinaNet CNN were located in paramediastinal positions, the U-Net CNN showed false negatives more uniformly distributed across the lung. Increased false negative rates along the mediastinum were already found in prior studies of blind-spot detection on CXRs^51–53^. The concentration of observer false negatives in paramediastinal positions could be due higher absorption coeffcients in this area and therefore less contrast. Another interesting observation in our study was that human readers did not fully utilise the 1-100 scale, but 86% of ratings where provided in increments of 10 (e.g. 10, 20, 30,...).

This study has some limitations: Above all, as stated before, the simulation does not completely resemble the real setting: The tumor shape generation was implemented using simple augmentation model, due to less implementation effort. Forward-projections were performed by a parallel beam projector, as cone-beam projectors are computationally more challenging. The radiograph resolution is further limited to 512 pixel width, as the CT scan resolution is not higher. Furthermore, while the test set was checked to be nodule-free, the potential presence of additional tumors in the training data set may impact the performance. However, as the true non-nodulous areas occur with a much higher frequency than falsely marked regions, this is probably compensated by the class imbalance effect^54^. Another limitation is the use of absorption values derived from HU values: here, future work could further improve the simulation model by using a polychromatic spectrum with different kVp settings. Moreover, a limitation is that the number of different nodules used in the study still was low. This number could be increased, in order to have more variation between the different nodules. Also, the number of healthy cases was low. As these were generated from healthy CT scans, only one X-ray image per scan was generated in order to avoid duplicate radiographs.

Since a simulation does never completely resemble the real setting, the radiologist’s performance may be slightly worse than an evaluation on real radiographs. Here, the expression of effects such as the silhouette sign or differences in the mediastinal area between upright and supine patient positions could play a role.

## Conclusion

In this study, we presented a framework that generated realistic looking radiographs by inserting nodules into existing CT volumes. The radiographs generated by the framework were used to train multiple CNNs and to evaluate the CNN performance against radiologists. We found our method to be adequate for initial CNN and observer performance evaluation. Thus, it could serve as an additional performance indicator for CNNs, as, contrary to manually annotated data, the groundtruth segmentations are perfectly accurate. Furthermore, our method allows to find positions where observers have problems identifying nodules: we found critical positions in paramediastinal positions.

## Supplementary Information

Supplementary Information.

## Data Availability

The .xlsx table file used for RJafroc evaluation is available as supplementary material. The Luna16 dataset used for training is available on^28^.
